# Resolving host-episymbiont interaction dynamics through continuous cultivation

**DOI:** 10.1128/msystems.00592-26

**Published:** 2026-09-28

**Authors:** Alex S. Grossman, Jacey Weng, Adam D. Silverman, Batbileg Bor

**Affiliations:** 1 Department of Microbiology, ADA Forsyth Institute 1816, Somerville, Massachusetts, USA; Boise State University, Boise, Idaho, USA

**Keywords:** episymbiont, Minisyncoccota, Patescibacteria, CPR, microbial dark matter, community dynamics, chemostat, bioreactor, community modeling, interbacterial interactions

## Abstract

Minisyncoccota are an elusive lineage of “microbial dark matter” predicted to compose ~25% of total bacterial diversity. Despite near ubiquity, these organisms are challenging to cultivate, resulting from their specialized episymbiotic lifestyle. All cultivated representatives, predominantly Nanosynbacteria, depend on cognate prokaryotic hosts for growth. Studying the growth dynamics of episymbiotic bacteria and their hosts in batch cultures suggests that episymbionts typically reduce host populations and that hosts eventually adapt to episymbiont stress after serial passaging. However, discontinuous batch cultures do not reflect natural interactions between these organisms due to their drastically different growth rates, which complicates the investigation of host inhibition and adaptation. To describe these dynamics, we utilized continuous culture via small-scale bioreactors. Within a bioreactor, host bacteria can be cultivated at a consistent growth rate, providing the perfect substrate for cultivation of model Nanosynbacteria. Quantification of time until host crash, crash severity, host adaptation, and stable co-culture population provides mechanistic ways to describe episymbiont-host interactions. We used these techniques to compare infection by three episymbionts, revealing distinct infection patterns ranging from mild inhibition with rapid host adaptation to rapid host collapse followed by “arms-race” oscillation dynamics. Then, bioreactors were used to quantify the episymbiotic role played by a known host-binding type 4 pili (T4P-2), demonstrating that loss of pilus-range host binding (approximately 100–800 nm) significantly delayed the host crash without altering general crash dynamics. These experiments reveal that episymbionts can have drastically different effects on bacterial communities and provide the tools necessary to describe strain/species differences and molecular interactions.

**IMPORTANCE:**

Episymbiotic Minisyncoccota represent one of the largest branches of life on Earth, as well as one of the least understood. Furthermore, because Minisyncoccota can manipulate their hosts’ growth and morphology, they have immense ecological potential to shape the communities they occupy, both environmental and microbiome-associated. Our study highlights, for the first time, the potential of small-scale continuous cultivation for studying episymbiotic interactions that cannot be captured in discontinuous cultures. Herein, we used these techniques to interrogate interspecies variation in host inhibition potential and to determine how loss of a pilus-dependent binding factor mechanistically alters the cycle of episymbiont infection; however, this cultivation platform will enable researchers to answer many new questions about these ubiquitous host-episymbiont interactions.

## INTRODUCTION

**Fig 1:**
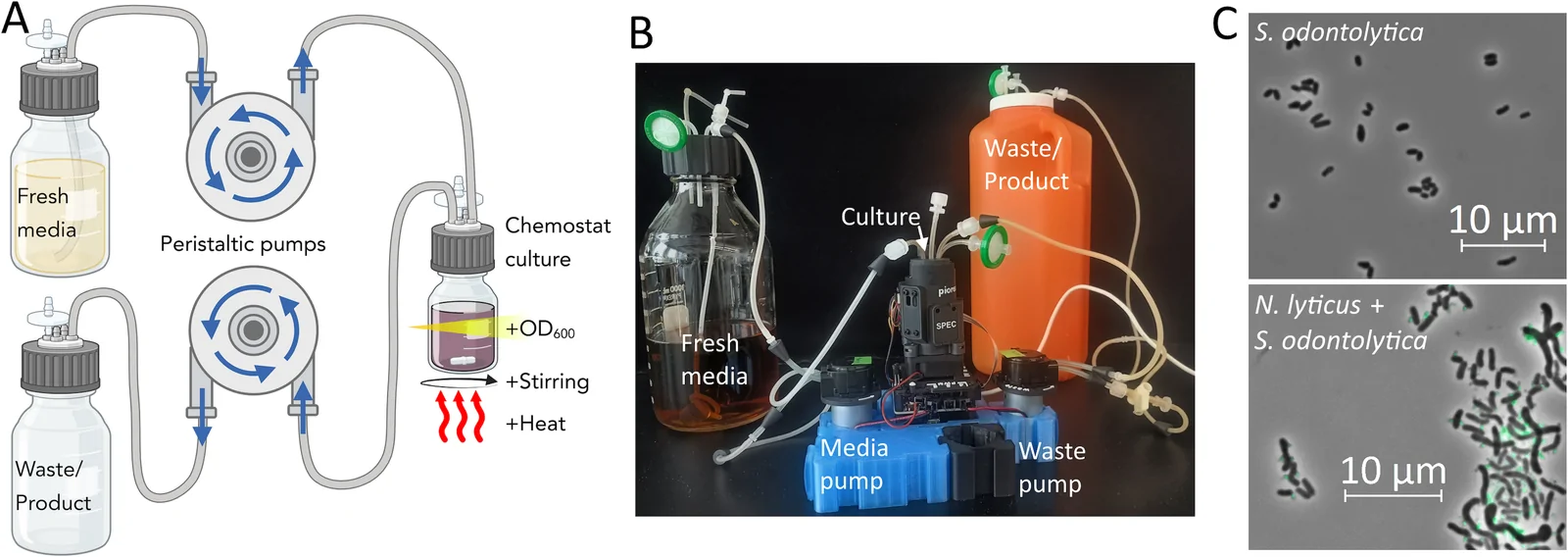
Cultivation of episymbiont co-cultures within a bioreactor. (**A**) A bioreactor diagram depicting the components required and the direction of flow. Created in BioRender. (**B**) Our experiments specifically adapted small Pioreactor platforms because of their size and modularity. These systems house the culture flask and sensors inside the central black tower that is flanked by the peristaltic pumps that power medium addition and culture removal. (**C**) Our model host bacterium, *S. odontolytica*, is native to the oral microbiome and typically grows as pleomorphic rods. When grown in co-culture with *N. lyticus* (expressing green fluorescent protein for easy visualization), the episymbionts attach themselves to *S. odontolytica* surfaces and induce host elongation and clumping.

Episymbiotic Minisyncoccota (formerly known as Patescibacteria or CPR) are elusive, ultrasmall microbes from an ancient branch of the bacterial family tree that have seemingly become an adaptive radiation specializing in obligate episymbiosis (1, 2). They possess streamlined genomes and grow in physical contact with the surface of larger bacteria or archaea (3). Very little is known about the ecological processes performed by these microbes (4). While microbial episymbiosis was discovered relatively recently, episymbiosis is not a rare lifestyle, having convergently evolved multiple times among bacteria (5, 6) and archaea (7, 8). Furthermore, the episymbiont-rich Minisyncoccota phylum contains approximately 25% of total bacterial diversity and a large portion of the “uncultivatable” microbial dark matter (9, 10). Within the last decade, several host-episymbiont co-cultures, predominantly oral isolates from the class Nanosynbacteria (formerly known as Saccharibacteria or TM7), have been successfully cultivated and used to demonstrate the profound ways episymbionts impact host microbes’ growth and morphology (2, 11, 12). These studies suggest that episymbionts exert powerful effects on microbiome function and composition, worthy of understanding. However, many bacteriological techniques are complicated by episymbionts’ host requirements. Growing two organisms with different reproductive tempos in a closed system prevents study of long-term population dynamics. Episymbionts grow significantly slower than their hosts, and episymbiont growth halts when the host reaches stationary phase (13, 14). Repeated subculturing of co-cultures, or passaging, can enrich episymbionts enough to detect host growth inhibition (12), and passaging beyond that point often results in episymbiont-resistant host adaptation (13). To understand the role of Nanosynbacteria within the human microbiome, we must understand the dynamics of host decline and adaptation; however, serial passaging makes these phenotypes intractable. Continuous cultivation in a carefully controlled bioreactor system with a steady flow of nutrients provides a more natural environment by enabling both populations to grow continuously (Fig. 1A) (15). This kind of bioreactor is referred to as a chemostat because it maintains a steady chemical composition via constant culture dilution. Furthermore, the recent development of small bioreactors, like the Raspberry Pi-powered Pioreactors used in this study (16, 17), reduces experimental costs and enables incorporation of chemostats into existing anaerobic/microaerophilic cultivation chambers capable of cultivation of Nanosynbacteria (Fig. 1B).

To probe these episymbiotic interactions, our experiments utilized a host organism isolated from the human salivary microbiome, the Actinomycete *Schaalia odontolytica* strain XH001 (Fig. 1C) (18). *Actinomyces*/*Schaalia* complex microbes are almost universally present commensals in the oral microbiome, occur at high abundance (19), and are often acquired at an early age (20, 21); however, they can also act as opportunistic pathogens contributing to Actinomycosis, blood infections, and polymicrobial infections (21, 22). Furthermore, studies of persistently inflamed tissues (periodontitis, gingivitis, and inflammatory bowel disease) show declines in *Actinomyces*/*Schaalia* populations and increases in the Nanosynbacteria that are predicted to use them as hosts, highlighting the complexity of these polymicrobial infection communities (23–27). *S. odontolytica* XH001 uniquely can host, or be infected by, three different cultivatable species from the family *Nanosynbacteraceae: Nanosynbacter lyticus* (HMT-952) strain TM7x, HMT-352 strain TM7-008 (ANI*_N. lyticus_* 83.8%), and HMT-488 strain BB004 (ANI*_N. lyticus_* 92.8%) (11, 13, 28). While all three episymbiont species share a common host organism and are isolated from the oral microbiome, previous research has shown that they induce different morphologies in host cells and differ in their competitive fitness (28). Specifically, TM7-008 is a strong competitor, BB004 is a weak competitor, and *N. lyticus* is intermediate. Additionally, to provide a reductionist example of how chemostat cultivation enables the study of episymbiosis, we used a previously identified *N. lyticus*Δ*pilA2* mutant that is unable to form host-binding type IV pili (28). Continuous cultivation enables analysis of how each episymbiont’s host infection cycle differs and how those differences impact host populations.

## RESULTS

**Fig 2:**
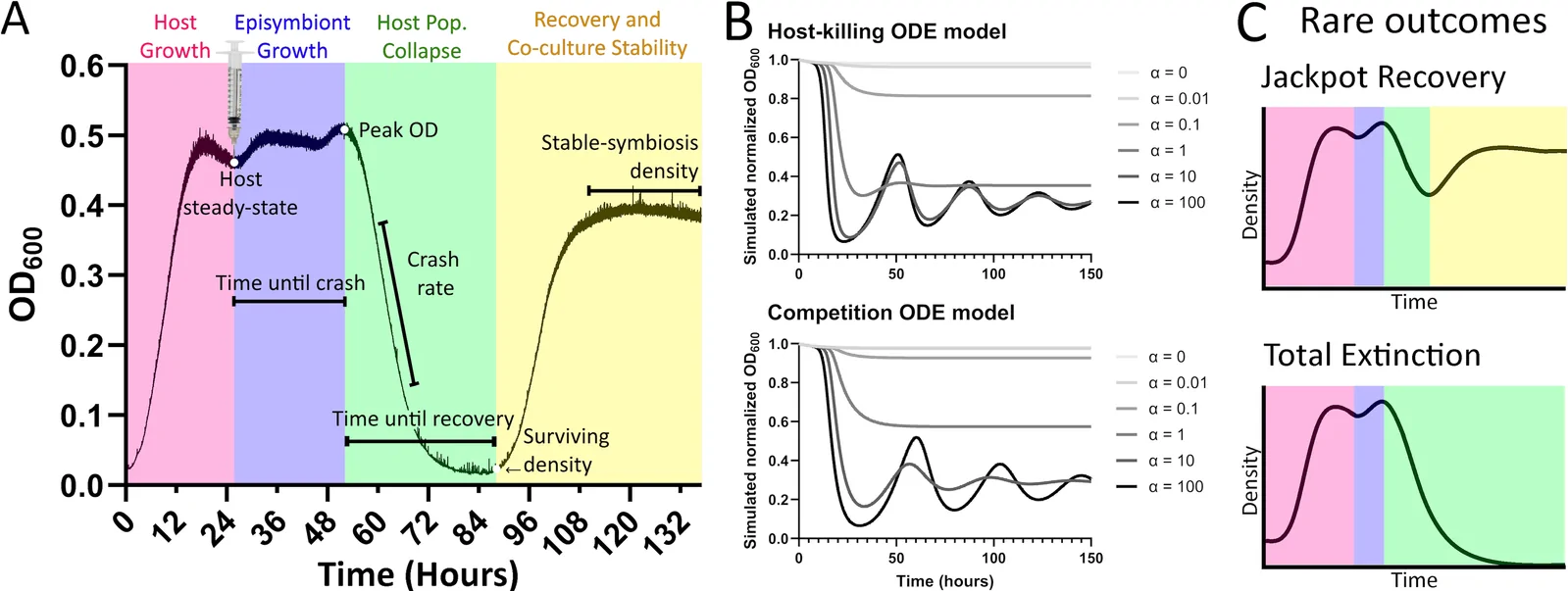
Model cycle of episymbiotic infection by a nanosynbacterium. (**A**) Schematic diagram of the standard episymbiont infection cycle as observed within a chemostat, divided into four phases; host monoculture growth until stability (pink), episymbiont growth on continuously growing host (blue), the collapse of the host population from episymbiont stress (green), and finally host adaptation/recovery (yellow). Important metrics such as OD_600_ values at phase transitions, time until crash, time until recovery, and crash rate are highlighted. (**B**) Two different ordinary differential equation (ODE) models were designed to simulate the observed cycles of infection; a host-killing model (top) that assumes episymbionts kill associated hosts at rate α and a competition-based model (bottom) that assumes episymbionts inhibit host growth at rate α. (**C**) Over the course of our experiments, two “rare outcomes” outside the standard models occurred. A jackpot recovery occurs when the host population adapts to become resistant before drastic growth inhibition, and a total extinction occurs when host growth is inhibited so strongly that all host cells are diluted out of the bioreactor or killed.

To interpret the cycle of episymbiont infection and host adaptation in continuous culture, new metrics had to be developed. First, host cells are grown until they reach a stable density and growth rate, resulting in a host steady-state (Fig. 2A). The host steady-state represents the point when the dilution factor of the chemostat matches the host monoculture’s growth rate. For our *S. odontolytica-*containing cultures, 15-mL volumes continuously fluxed media at a flow rate of 4.56 mL/h, resulting in a dilution factor of 0.30/h. This dilution rate worked for all species tested; however, this technique may not be compatible for episymbionts with growth rates many times slower than those of their hosts. To visualize the impact of episymbionts on host growth after infection, the host steady-state was used to normalize all later OD_600_ readings (Fig. S1A and B). Next, isolated episymbionts (~4 × 10^7^ cells) were injected into the bioreactor at roughly a multiplicity of infection (MOI) of 0.01 episymbionts/host. For our purposes, episymbionts are assumed to have no impact on optical density because their light scattering is negligible relative to host cells (14). After infection, densities either remained constant or showed slightly enhanced growth for a number of hours, which we termed “time until crash.” Eventually, episymbionts begin inhibiting host growth, causing the optical density to start a precipitous, logarithmic decrease, which we termed the crash phase. Two metrics were used to quantify crash phase severity: crash rate was determined by fitting a linear function to the period of peak decline, and surviving population was estimated using the lowest density achieved before population recovery began. Eventually, host adaptation and dilution of episymbionts allow host populations to begin increasing, resulting in a recovery phase. The time between crash and host recovery was termed “time until recovery” and used as a proxy for rate of host adaptation. After the recovery phase, host-episymbiont co-cultures eventually reach a new steady-state termed stable-symbiosis density, at a lower OD_600_ than the host steady-state (Fig. 2A).

In parallel with our experiments, we developed two ODE models to qualitatively reproduce the host-episymbiont growth dynamics observed in our experiments (see Supplemental material for model details). Our first model assumes that the episymbiont actively kills the host (with second-order rate constant α) and that the episymbiont growth rate is proportional to the host population, like a Lotka-Volterra model (Fig. 2B; Supplemental material, equations 1 to 6). Our second model instead assumes that episymbionts compete with hosts for resources and inhibit host growth (at inhibition coefficient α) without directly killing them (Fig. 2B; Supplemental material, equations 1, 2, and 7 to 10). Both models produced similar simulated infection cycles, showing that changes to the α parameter drastically altered the host crash rate, surviving host population, and stable-symbiosis density. Models also predicted that sufficiently high α values could cause long-term oscillations in host density that gradually dampen in amplitude. Since it is unclear if episymbionts kill their hosts, inhibit host growth, or some combination of the two, we consider both models in our studies.

During our experiments, we observed two rare outcomes that could happen outside a continuum ODE model: jackpot recovery and total extinction (Fig. 2C). Jackpot recovery occurs when the host population manages to adapt before completely crashing, presumably due to a mutation before or during infection. Conversely, total extinction occurs when the host population fails to adapt before continuous dilution removes them from the population completely. Each rare outcome only occurred once or twice during our studies to date (41 total runs), and as such, their true frequencies cannot yet be estimated. Notably, neither rare outcome would have been detectable in experiments using discontinuous, serial passaging cultivation.

**Fig 3:**
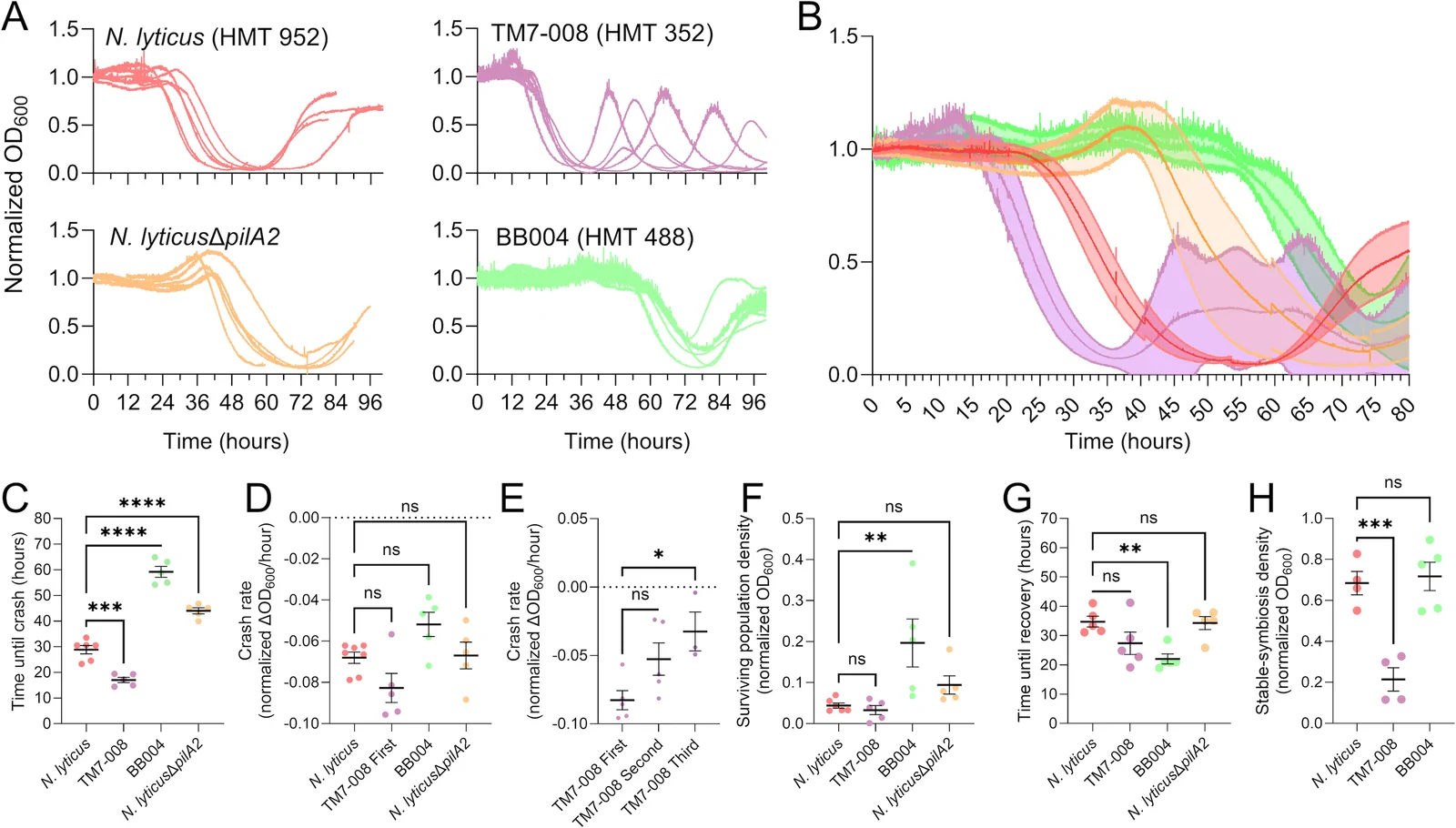
Population dynamics of constantly growing *S. odontolytica* infected by diverse episymbionts. (**A**) Continuous cultivation of co-cultures infected by *N. lyticus* (red), *N. lyticus*Δ*pilA2* (yellow), TM7-008 (purple), and BB004 (green) demonstrates distinct infection cycles and host inhibition intensities, most clearly visible when (**B**) averaging 5 or 6 replicates. Most strains induce host inhibition, cause a population collapse, become subject to washout, and then recover to a stable state; however, TM7-008 induces two or more sequential crashes/recoveries before the host can adapt. To quantify episymbiotic impact on host populations, curves were analyzed for (**C**) time until crash, (**D**) maximum crash rate (comparing species), (**E**) maximum crash rate (comparing sequential crashes), (**F**) lowest surviving population density achieved during crash, (**G**) time until recovery, and (**H**) stable symbiosis density after recovery. The time until crash metric showed the most sensitivity and consistency between replicates. (**C–H**) Statistical significance was determined via one-way ANOVA with Dunnett’s comparison. ns, not significant; *, *P* ≤ 0.05; **, *P* ≤ 0.01; ***, *P* ≤ 0.001; ****, *P* ≤ 0.0001.

Experimentally, *Nanosynbacteraceae* species’ infection cycles differed significantly, revealing a continuum of host-inhibition severity and the potential for oscillating population booms and busts (Fig. 3A and B). *N. lyticus* (shown in red) and BB004 (green) both followed the standard infection cycle depicted in Fig. 2A. Conversely, TM7-008 infected populations (purple) crashed and recovered two or more times, with amplitude dampening between each subsequent crash, exactly as our ODE models predicted would occur for strains with a high α value. *N. lyticus* induced host population crash ~30.0 h post-infection (hpi), TM7-008 induced its first host crash at ~17.1 hpi, and BB004 induced host crash at ~59.2 hpi (Fig. 3C). This pattern perfectly matches the pattern of competitive fitness previously observed in these strains (28), suggesting that the rate at which an episymbiont can establish host association/inhibition may dictate their competitive potential.

Observed crash rates had wide intra-treatment variance and, despite having trends that resembled time until crash, did not significantly differ between species (Fig. 3D; Fig. S1C thorugh E, S2A). However, during infection by TM7-008, the “later” crashes had a significantly decreased crash rate relative to the initial host crash (Fig. 3E), reflecting the gradual amplitude dampening. During the crash phase, populations infected by *N. lyticus* and TM7-008 had surviving population densities between 3% and 4% on average, while those infected by BB004 had a larger average surviving population of 19.7% (Fig. 3F; Fig. S2B). Furthermore, BB004-infected populations had a significantly shorter time until recovery (22.0 h post-crash) than those infected by *N. lyticus* (34.8 hpc) (Fig. 3G; Fig. S2C). These data suggest that BB004 is either poorly adapted to parasitize *S. odontolytica* XH001, or it has adapted to be less parasitic/more commensal than the other assayed species. Like the crash rate, the recovery rate was approximated by linear regression; however, all strains showed similar recovery rates (0.04–0.06 normalized OD_600_/h), suggesting it is intrinsic to the basal growth rate of *S. odontolytica* and the dilution factor of the chemostat (Fig. S1G). After population recovery, *N. lyticus* and BB004 co-cultures reached similar stable-symbiosis densities at 67%–68% of the host steady-state (Fig. 3H). Due to constant oscillation, most TM7-008 co-cultures did not reach a true stable-symbiosis density before the experiment ended, so stable-symbiosis densities were estimated by averaging their last 600 readings (50 min). After two or three crash/recovery oscillations, TM7-008 co-cultures approached a significantly lower stable-symbiosis density, at ~21%, suggesting that TM7-008 may be more parasitic/less commensal than the other assayed species. This experimentally observed shift in stable-symbiosis density favors the host-killing ODE model because the competition model predicts that stable-symbiosis density would only vary for strains with low α values (Fig. S2D).

To better understand how a single mechanistic change can impact the episymbiont infection cycle, we utilized an *N. lyticus* mutant that is defective in host binding because it cannot produce one of the two type IV pili produced by *N. lyticus*. In previous studies (28), passage-based cultivation of *N. lyticus*Δ*pilA2* demonstrated reduced growth and host binding in early passages that, with additional passages, could eventually reach high concentrations like those of the wild-type. We hypothesized that this strain would have an elongated time until crash but would otherwise be similar to the wild type. Within our bioreactors, *N. lyticus*Δ*pilA2* did induce a significant delay in time until crash (45.0 hpi) relative to the wild type (30.0 hpi) (Fig. 3A through C), and despite this 50% delay in establishment, the crash rate and time until recovery were unaffected (Fig. 3D through G). This establishes that the time until crash metric is influenced by how long it takes an episymbiont to find and bind its host, while the other infection cycle dynamics appear to be independent phenomena. Unfortunately, stable-symbiosis density values were not determined for *N. lyticus*Δ*pilA2* replicates due to the significant crash delay.

**Fig 4:**
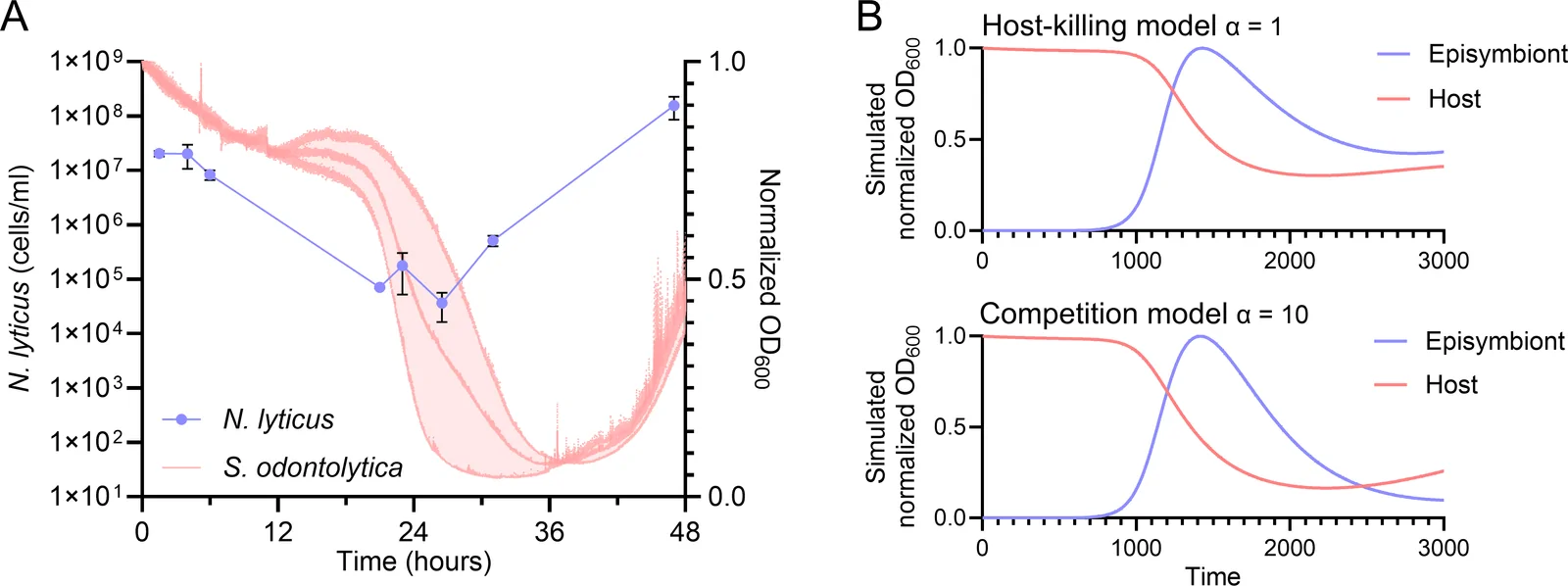
Enumeration of episymbionts during and after host crash. Manual subsampling of spent media from bioreactors at critical time points enables qPCR enumeration of episymbionts. (**A**) Overlaying episymbiont and host populations (approximated via optical density) shows minimal episymbiont growth until the midpoint of the host crash phase, and extensive growth after the crash or during recovery. Conversely, (**B**) when using the lowest α values that induced host crash, the host-killing and competition ODE models predicted that episymbiont growth would be highest before the crash phase. The models disagreed on episymbiont populations during host recovery, with host-killing predicting high abundances (as observed experimentally) and competition predicting low abundances.

Next, to investigate episymbiont population dynamics during the crash phase, host cultures infected with *N. lyticus* (MOI = 0.1) were manually subsampled for quantitative polymerase chain reaction (qPCR) enumeration of episymbionts (Fig. 4A). Prior to the crash phase, both the episymbiont and host populations showed a steady decline, suggesting that both populations were growing more slowly than they were diluted. However, the episymbiont population appears to stabilize around the midpoint of host crash and then significantly increase prior to host recovery. This population boom of episymbionts post-crash is consistent with the episymbiont/host ratios previously observed in discontinuous cultures (14). Conversely, both ODE population models predicted that episymbiont populations would peak early in the crash phase because these models assume that episymbiont density drives host inhibition (Fig. 4B). This disparity suggests that host inhibition is achieved before episymbiont populations surge, potentially through molecular disruption of host processes. The host-killing ODE model predicts that episymbionts remain numerous post-crash, while the competition model predicts that host populations will only recover after episymbionts are mostly removed via dilution (Fig. 4B). In this sense, the experimental data more closely resemble those of the host-killing model since they show episymbionts remaining numerous through host recovery.

**Fig 5:**
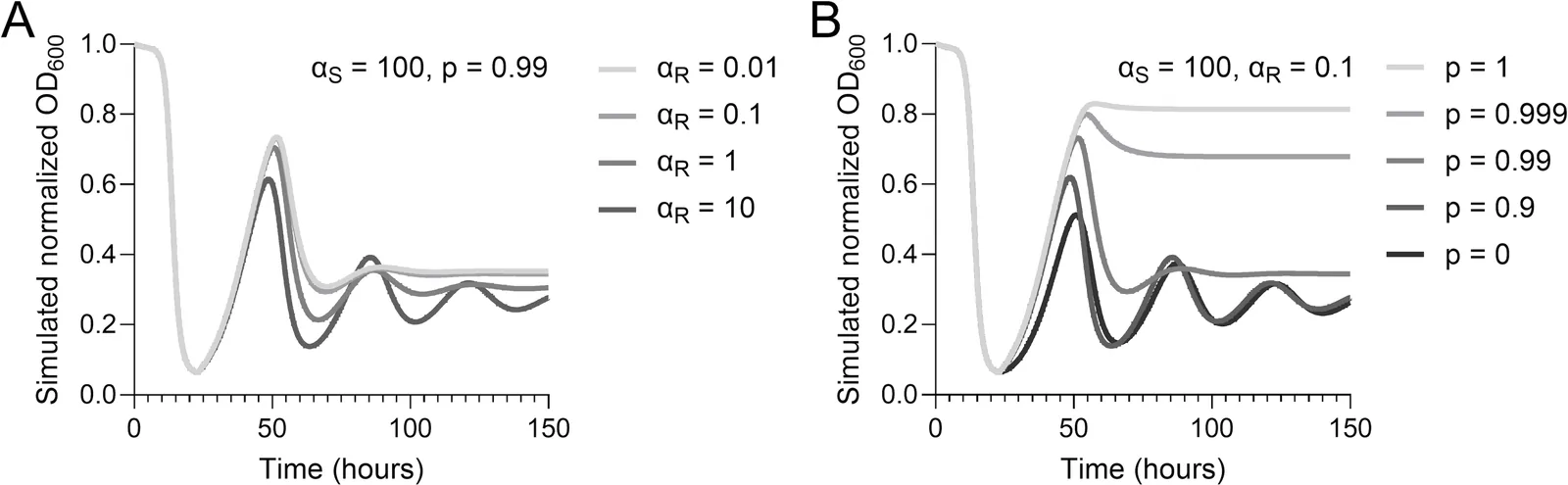
A two-host ODE model simulating selection for episymbiont resistance. Like in the host-killing episymbiont model, episymbiont-susceptible hosts are initially inhibited at rate α_S_; however, after the crash-susceptible hosts become resistant at rate p. Resistant hosts are not immune to episymbiont inhibition but are inhibited at the lower rate α_R_. (**A**) Simulating infection cycles while varying the α_R_ parameter shows that oscillations do not occur for sufficiently resistant host cells. (**B**) Holding α_S_ and α_R_ steady and varying the p parameter predicts that the percentage of hosts that acquire resistance strongly influences stable-symbiosis density. Furthermore, population oscillations are not predicted to occur when susceptible hosts are absent (*P* = 1) or rare (*P* > 0.99).

Both of our ODE models predict that, for large values of the α parameter, the host population crashes to a small fraction of host steady-state and then undergoes decaying oscillation to the stable-symbiosis density (Fig. 2B). However, the wild-type *N. lyticus* infection cycle shows a different dynamic, in which the host population crashes to a small community of survivors and then nearly recovers to the host steady-state without oscillation (Fig. 3A). We hypothesized that a model that incorporated host adaptation could explain this dynamic. If a population of parasite-resistant hosts are selected for, then the population’s α parameter value decreases post-crash. We simulated this *in silico* using a modified version of the host-killing ODE model that introduced a probability (p) that hosts would become resistant post-crash (see Supplemental material for model details). In this two-host (“resistant” and “susceptible”) model, the rebound population tends to stabilize without oscillation (Fig. 5A). When varying the probability of host resistance, the two-host ODE model predicts that stable-symbiosis density correlates to the percentage of resistant hosts and that oscillation only occurs when an episymbiont’s α parameter is high and few resistant hosts emerge after the crash phase (Fig. 5B).

## DISCUSSION

Studying episymbiont-host interactions intrinsically poses many technical challenges. These studies have focused on a single host organism and a small panel of episymbionts to develop new techniques and to quantify the ecological impact of different species and genotypes. The resulting data demonstrate that even closely related episymbionts can have distinct impacts on host population dynamics. The most statistically impacted and replicable metrics were (i) time until crash and (ii) stable-symbiosis density (Fig. 3C through H), suggesting that population dynamics may be disproportionately impacted by episymbionts’ rate of binding/establishment and the capacity of the host to adapt to an episymbiont’s presence. The importance of the host binding rate was emphasized by the large host crash delay observed in the binding deficient T4P mutant. Concurrent tracking of episymbiont and host populations suggests that episymbiont growth is relatively low during this establishment and high during the latter half of the host crash, suggesting that crash initiation does not require overwhelming episymbiont abundance. Using this new toolset, we observed that Nanosynbacteria can exist across the commensal-to-parasite continuum, but the potential for continuous cultivation to elucidate the microbial ecology of episymbiosis does not stop here. Future experiments could utilize mock communities to quantify realistic episymbiont competitions, perform continuous evolutionary experiments under changing conditions, or screen other *N. lyticus* mutants to better understand the physiological features that shape the host-inhibition factor α. A major limitation of our current experimental setup is our limited capacity to quantify the episymbiont and host populations simultaneously. Future experiments could implement episymbiont tracking directly, by incorporating an on-bioreactor fluorometer and using fluorescent protein-expressing episymbionts, or indirectly, by incorporating a fluidic autosampler and using downstream qPCR analysis to quantify episymbionts with fine-grained resolution. Having real-time tracking of both populations would significantly improve our ability to model host-episymbiont dynamics. Due to the limited availability of episymbiont strains, this study exclusively examines species within the class Nanosynbacteria. If future studies could establish continuous cultivation of other Minisyncoccota organisms, or episymbiotic archaeans, a more comprehensive picture of this ecology could be constructed.

The ODE models described here are helpful for translating how episymbionts with different inhibitory potentials affect their host population; however, no model perfectly captured the dynamics of all strains. Since our studies highlighted the importance of host adaptation to co-culture outcomes, we developed two-host models (susceptible and resistant), which resembled our experimental results better than single-host models. However, these models compress the complicated process of adaptation to a few variables, and realistically, far more data would need to be collected to determine the modes of inhibition utilized by episymbiotic species and the process of host adaptation. These models can be refined, rethought, or even combined as infection cycles from more host-episymbiont pairings are quantified. Simply by providing a continuous supply of new hosts, a more realistic environment can be recreated, and episymbiont behaviors can be made experimentally tractable in ways they never have been before.

## MATERIALS AND METHODS

### Bacterium cultivation conditions

*S. odontolytica* XH001, *N. lyticus* TM7x, *N. lyticus* NS1::sfGFP (29), TM7-008, and BB004 strains were cultivated in brain heart infusion (BHI) broth (Fisher: 237200) at 37°C under microaerophilic conditions (5% CO_2_, 2% O_2_, and 93% N_2_) in either a Whitley A35 or Coy chamber. Prior to chemostat cultivation, *N. lyticus* cells were isolated as previously described using 0.45-µm filters (14). All episymbionts were isolated from existing *S. odontolytica-*containing co-cultures (18, 28). Bacterial cultures were stored at −80°C in 15% glycerol cryo-protectant.

### Chemostat operation

All experiments were performed using four Pioreactor 20-mL chemostats (hardware v1.0 and v1.5; software v25.11.19) (Pioreactor Inc. Waterloo, Canada) (16, 17). Prior to chemostat operation, the reactor vial, tubing, medium bottles, and waste receptacles were cleaned by cycling through 50 mL of 2% bleach and 50 mL of ultrapure water. Bleaching was essential for these experiments because minute *S. odontolytica* biofilms within the tubing can survive autoclave sterilization alone. A volume of 700 mL of BHI medium was added, and then materials were autoclaved for sterility and assembled in a biosafety hood. Then, 300 μL of overnight *S. odontolytica* culture was sterilely inoculated into the reactor via syringe, and BHI was added to reach the vial’s working volume of 15 mL. A volume of 380 μL of fresh medium was pumped into the chamber every 5 min to maintain a 0.30 dilution rate. Host cells were grown continuously for 24–36 h to reach a steady-state OD_600_ and growth rate. Upon reaching stability, 200 μL of isolated TM7x at OD_600_ 0.005 (MOI = 0.01 for interspecies/strain comparisons) or OD_600_ 0.05 (MOI = 0.1 for qPCR quantification of episymbionts) was injected into the reactor. Pioreactors were programmed to maintain a temperature of 37°C, spin the culture at ~500 rpm, and take OD_600_ measurements every 5 s. Continuous cultures were maintained until cultures completely collapsed and, where possible, allowed to continue further into the recovery phase. All interspecies/strain comparisons were replicated 5 or 6 times. Episymbiont quantification was replicated three times. Subsampling was performed via sterile extraction from bioreactor waste lines, and *N. lyticus* was quantified using previously published primers (TM7x_F, tcgaagagcagaaacgcaca; TM7x_R, tcttttgccttcgcctcagt) (28). After experiment conclusion, all cultures were assessed for potential contamination via phase contrast microscopy (Nikon Eclipse E400). Data were normalized to host steady-state OD_600_ to account for discrepancies between Pioreactor sensors. All data were analyzed in GraphPad Prism 10 (Version 10.3.0) via one-way analysis of variance with Dunnett’s comparison. Crash rate and recovery rate were calculated via linear regression of the 6-h period of maximum decrease/increase. A detailed step-by-step version of this protocol is available on protocols.io at https://dx.doi.org/10.17504/protocols.io.14egn5r9yg5d/v1.

### Computational modeling

All details and equations for the host-killing and nutritional-competition ODE models are included in the supplemental material. Simulations were calculated in Python version 3.14. Code is available at https://github.com/grossmanmicrobio/Continuous-Cultivation-EpiModels.

## Data Availability

All data from the experiments described are available from the corresponding author upon request. All codes used to simulate interbacterial dynamics are available at https://github.com/grossmanmicrobio/Continuous-Cultivation-EpiModels.

## SUPPLEMENTAL MATERIAL

Supplemental material msystems.00592-26-s0001.docx Model definitions and supplemental figures.

ASM does not own the copyrights to Supplemental Material that may be linked to, or accessed through, an article. The authors have granted ASM a non-exclusive, world-wide license to publish the Supplemental Material files. Please contact the corresponding author directly for reuse.
